# Next-generation microbiome therapeutics: psychobiotics and fecal microbiota transplants for mental health treatment

**DOI:** 10.3389/fcimb.2026.1719357

**Published:** 2026-08-28

**Authors:** Daniel Raja Femil Selta, Leggins Abraham, Rangasamy Kavitha, Kongkona Saikia, Abiram Karanam Rathankumar, Monica Mironescu, Ion Dan Mironescu, Chella Perumal Palanisamy, Gopalakrishnan Velliyur Kanniappan

**Affiliations:** 1 Department of Biochemistry, Sri Ramakrishna College of Arts and Science for Women, Coimbatore, India; 2 Department of Physics, Sri Ramakrishna Engineering College, Coimbatore, India; 3 Department of Biochemistry, Bharathiar University, Coimbatore, India; 4 Department of Biochemistry, Faculty of Arts Science Commerce and Management (FASCM), Karpagam Academy of Higher Education, Coimbatore, India; 5 Department of Biotechnology, Faculty of Engineering (FoE), Karpagam Academy of Higher Education, Coimbatore, India; 6 Faculty of Agricultural Sciences Food Industry and Environmental Protection, Lucian Blaga University of Sibiu, Sibiu, Romania; 7 Phytochemistry and Pharmacotherapy Lab, Department of Dermatology, Saveetha Medical College & Hospital, Saveetha Institute of Medical and Technical Sciences (SIMATS), Chennai, India; 8 Natural Products and Cancer Biology Lab, Department of Physiology, Saveetha Medical College & Hospital, Saveetha Institute of Medical and Technical Sciences (SIMATS), Chennai, India

**Keywords:** anxiety, depression, dysbiosis, mental health, microbiome

## Abstract

The human gastrointestinal tract (GIT) harbors a diverse microbial community, collectively referred to as gut microbiota, which plays an essential role in maintaining host physiology, metabolism, immune function, and neurobehavioral processes. Mounting evidence highlights the importance of the bidirectional Gut–brain (GB) axis, through which gut microbes influence neural signalling, stress response, and emotional regulation. Dysbiosis of this axis has been associated with psychiatric, neurodevelopmental, and neurodegenerative disorders, such as anxiety, autism spectrum disorder (ASD), major depressive disorder (MDD) and schizophrenia. A growing area of research has identified psychobiotics—live microorganisms with psychotropic potential—as promising therapeutic agents for mental health conditions. These microbes exert their effects through multiple mechanisms, including modulation of neurotransmitter production, short-chain fatty acid (SCFA) signalling, immune regulation, and hypothalamic–pituitary–adrenal (HPA) axis stabilization. Preclinical and clinical studies provide supportive evidence for their antidepressant and anxiolytic effects, although large-scale, long-term trials remain limited. In parallel, fecal microbiota transplantation (FMT) has emerged as a potential strategy to restore microbial balance and improve psychiatric symptoms. Early findings demonstrate its role in modulating immune pathways, such as NLRP3 inflammasome signalling, and neurotrophic factors via gut microbial reshaping. Together, psychobiotics and FMT represent next-generation microbiome therapeutics that may complement conventional psychiatric interventions. This review synthesizes current evidence and highlights the future potential of microbiome-based strategies in treating mental health disorders.

## 1 Introduction

The human gastrointestinal tract (GIT) harbors a wide range of microorganisms which are commonly called ‘gut microbiota’. They primarily consist of bacteria; however, viruses, protozoa, fungi, and archaea are also present (Sender et al., 2016). When compared to the total human DNA, the collective microbial genome is very high, such that for every human gene, there are over 100 bacterial genes (Backhed et al., 2005). Considering their enormous genetic potential, these microbes play important roles in almost all physiological processes. Thus, gut microbiome can be considered the key regulator of host physiology, influencing metabolism, immune function, and also neurobehavioral processes.

Recently, various studies have been conducted on the bidirectional communication system between the gut and the brain, known as the microbiome–gut–brain (MGB) axis. Through this axis, microbes influence brain development, stress management, mood regulation. Thus, any imbalance in the gut microbiota can directly influence psychiatric and neurodevelopmental disorders. This influence is well-documented in various psychiatric illnesses. Some of them include appetite and weight changes in major depressive disorder (MDD) (Privitera et al., 2013), and, diarrhoea and nausea in anxiety disorders (Mussell et al., 2008). Gastrointestinal problems are common in neurodevelopmental and psychiatric disorders. The prevalence of gastrointestinal symptoms is reported to be 82.4% in autism spectrum disorder (ASD) (Leader et al., 2021, 2022), 30% - 60% in schizophrenia (Nagamine, 2025), and 60% in Parkinson’s disease (Poole et al., 2011; McElhanon et al., 2014; Severance et al., 2015). The brain and GIT maintain a two-way communication system that continuously participates in signalling pathways. The gut microbiome also modulates key neuroendocrine and neurotransmitter systems — including the hypothalamic-pituitary-adrenal (HPA) axis and serotonergic, dopaminergic, and GABAergic pathways — that are closely linked to mental health (Rea et al., 2016; O’Mahony et al., 2015). The mechanistic basis of these interactions is discussed in detail in subsequent sections.

In recent years, the field of MGB axis has moved forward from merely describing a relationship between dysbiosis and mental illnesses to studying more mechanistic and translationally useful models. Current research is more focused on microbial metabolome profiling, immune communication between hosts and microorganisms, neuroinflammatory effects caused by inflammasomes, and functional relevance of specific strains or consortia of microorganisms as determining factors of neurobehavioral phenotype. These innovations have moved the field past the scope of classic probiotic approaches to newer and more advanced microbiome-related therapies, such as psychobiotics, postbiotics, microbial consortia, and improved fecal microbiota transplantation (FMT). All of the above can be described as a shift towards precision medicine in microbiology, as microbial ecology, functional pathways, metabolomics, and host inflammation will ultimately determine the course of future mental disease treatment (Bautista et al., 2026; Yaqub et al., 2025; Ķimse et al., 2024).

With an increasing interest in microbiome-based strategies for mental health disorders, the current review focuses on the possibility of psychobiotics and FMT as next-generation microbiome therapeutics as psychiatric interventions. The development of this narrative review article has been carried out through a structured search of the scientific literature, using PubMed, Scopus, Web of Science, and Google Scholar databases. The relevant literature has been searched using a combination of the following terms: “gut microbiota”, “gut-brain axis”, “microbiome-gut-brain axis”, “psychobiotics”, “probiotics”, “fecal microbiota transplantation”, “FMT”, “depression”, “anxiety”, “autism spectrum disorder”, “schizophrenia”, “stress”, “neuroinflammation”, “short-chain fatty acids” and “mental health.”

The search was conducted mainly on English-language peer-reviewed journals with published preclinical research, clinical trials, mechanistic research, and highly relevant review articles on the topic of microbiome-based modulation of psychiatric and neurobehavioral outcomes. Emphasis was given to research on the topics- microbial metabolites, immune and inflammatory signaling pathways, regulation of neurotransmitters, HPA axis modulation, and microbiome-based therapeutic approaches including psychobiotics and FMT. The literature searched was mainly published between 2005 and 2026, although landmark publications published before this period were included which were appropriate to establish a background and conceptual understanding of the topic.

## 2 The gut-brain axis: a bidirectional communication

The gut microbes can impact the structure, function, and development of the brain through neuroimmune and neuroendocrine mechanisms (Martin et al., 2018). Interoception, which refers to sensing the body’s internal physiological state, is crucial to mood changes, emotional responses, and homeostatic reflexes (Mayer et al., 2006; Bonaz et al., 2021). The important function of these reflex loops is to sustain homeostasis in the gut-brain (GB) axis during stable conditions and disturbances (Carabotti et al., 2015). This mechanism of these reflexes is mostly influenced by the autonomic nervous system (ANS) during a change in the body homeostasis (Mayer and Tillisch, 2011). In this context, gut microbiota plays a major role and produces signals that affect the body in two main ways. First, they can act locally by influencing enteric neurons or by stimulating nerve endings in the vagus and sympathetic nervous systems, which send internal body signals up to the brain. Second, these signals can reach the brain through the bloodstream.

**Figure 1:**
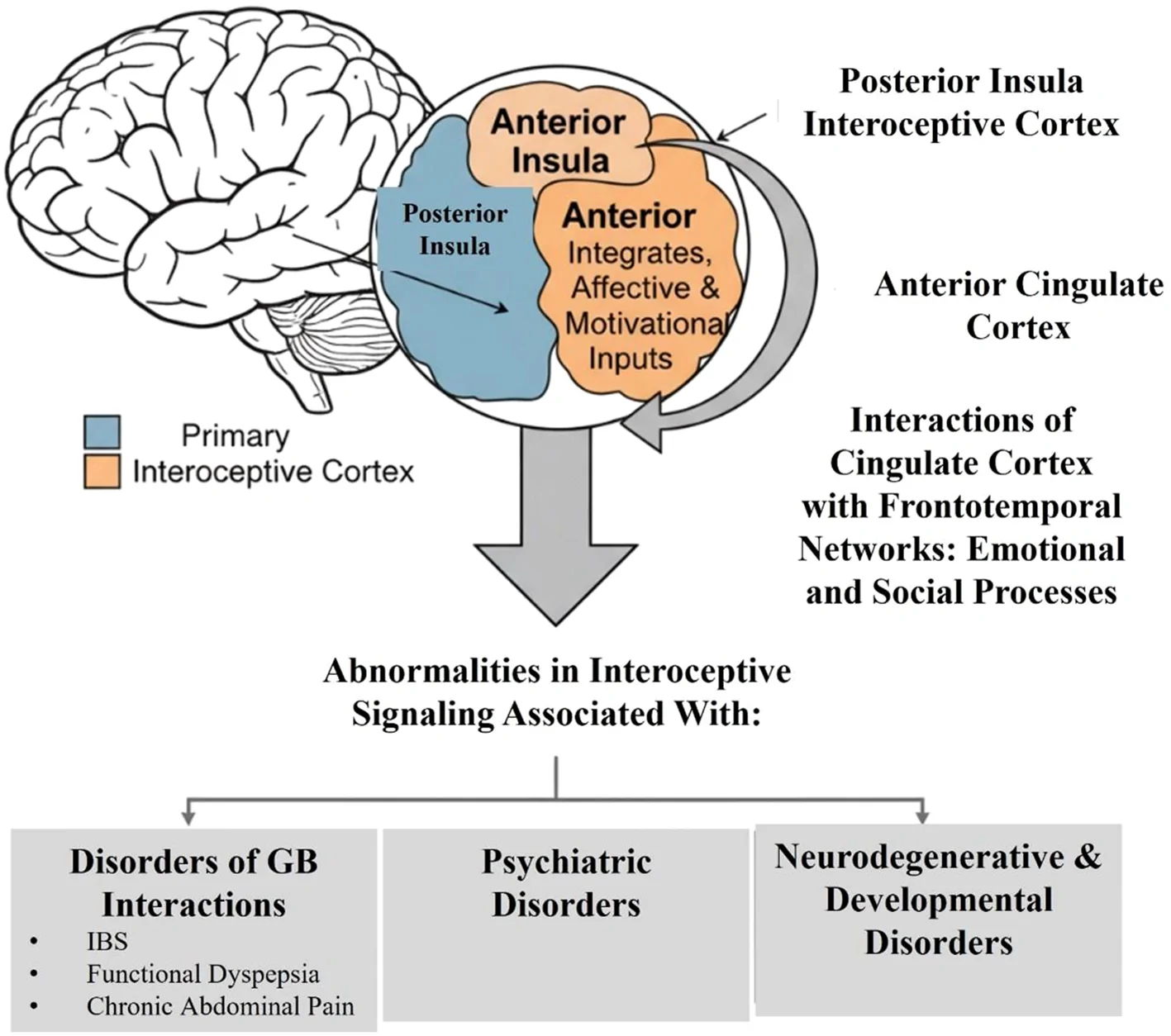
Proposed role of insular cortex dysfunction and altered interocep tive processing across gastrointestinal, psychiatric, neurodegenerative, and neurodevelopmental disorders. This schematic summarizes the hypothesized mechanisms by which structural and functional abnormalities of the insular cortex may disrupt interoceptive processing and contribute to disease pathophysiology across multiple clinical conditions. As a central node in the gut-brain axis, the insular cortex integrates visceral, autonomic, affective, and cognitive inputs to generate subjective bodily awareness and adaptive physiological responses. Dysregulation within this network may lead to impaired visceral signal interpretation, altered autonomic and emotional processing, and maladaptive brain-body interactions, thereby contributing to symptom generation and progression in gastrointestinal, psychiatric, neurodegenerative, and neurod evelopmental disorders.

Generally, these signalling molecules fall into three groups: metabolites derived from the food we eat, compounds produced within the body itself, and components that come from the microbial cell walls (Needham et al., 2020). Signals derived from microbes can reach the central nervous system (CNS) either by systemic circulation or by receptor interaction. In response, the CNS modulates the sympathetic and parasympathetic activities of the ANS and also influences the HPA axis (Yano et al., 2015). Any changes in these interoceptive signals can cause perturbations. Significantly, the MGB axis is not merely a set of discrete signaling pathways, but rather a holistic system-level network, wherein the interactions between microbial metabolites, the integrity of the epithelial barrier, immune signaling, neuroendocrine signaling, and host cell programs are constantly occurring. Under such an understanding, the impact of gut bacteria on the brain is not limited to their actions via the signaling pathway of neurotransmitters. They create a biological context by affecting the permeability of the intestines, inflammatory mediators, vagus nerve signaling, endocrine stress reactions, and metabolite levels. Such pathways are highly interrelated, and any disruption at one level—for instance, in microbial metabolite levels or the integrity of the barrier—can lead to a cascade of effects at the level of neuroimmune and neurobehavioral function. The modern scientific literature has increasingly embraced a holistic approach to the gut-brain (GB) axis, such that any microbiome-related psychiatric phenomena are more accurately understood as host-microbe network interactions rather than singular signaling pathway phenomena (Verma et al., 2024; Lee et al., 2025; Singh et al., 2024). Further, deviations in these signalling can generate symptoms in GB interaction disorders like IBS, functional dyspepsia, chronic abdominal pain, psychiatric disorders, neurodegenerative and developmental disorders (**Figure 1**).

**Figure 2:**
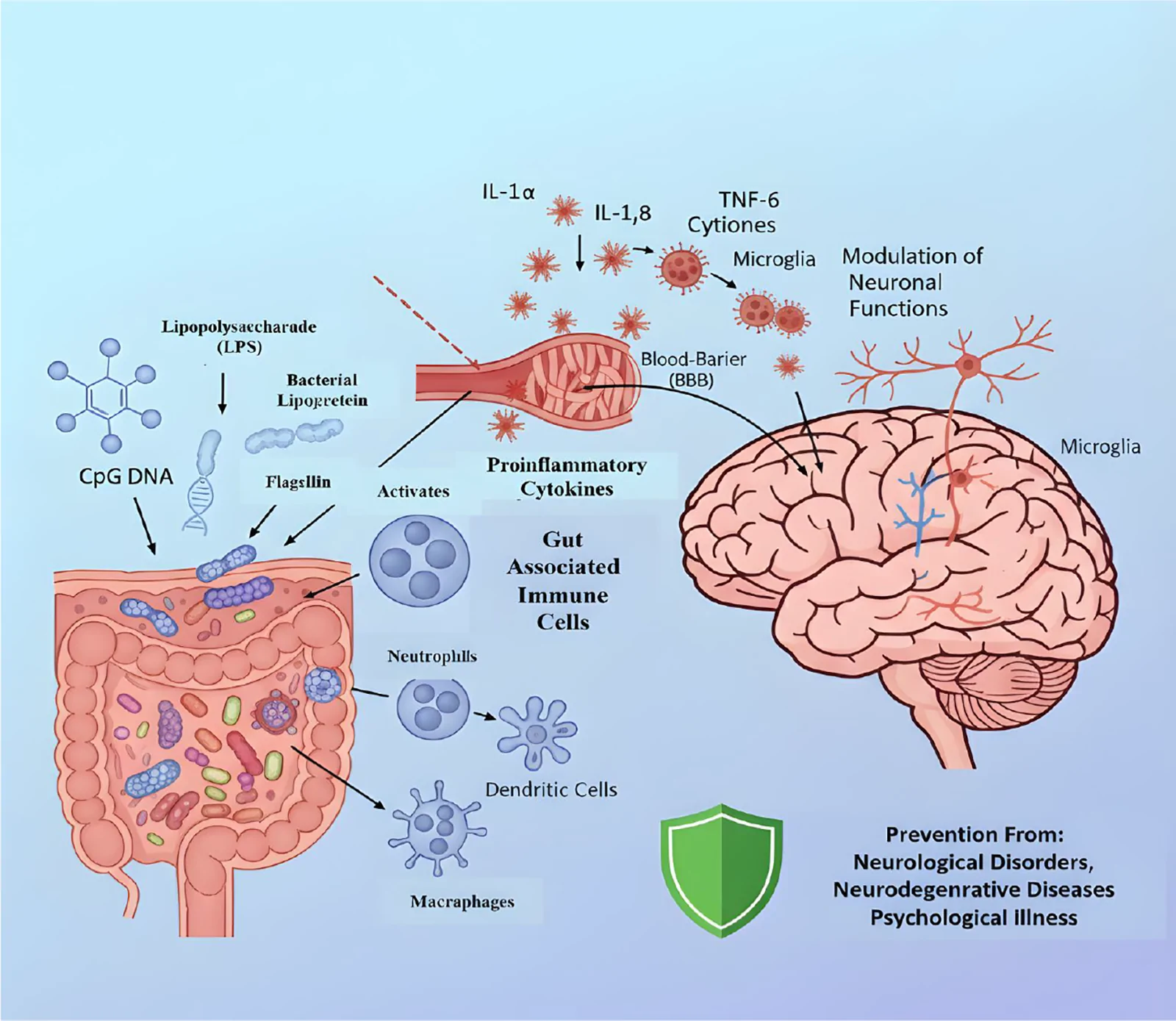
Proposed mechanisms linking gut-brain axis dysregulation to psychiatric, neurodegenerative, and neurodevelopmental disorders. This schematic illustrates the role of the gut-brain axis as a bidirectional communication network connecting the gut microbiota with the central nervous system via neural, immune, endocrine, and metabolic signaling pathways. Gut microbial dysbiosis may induce the production of pro-inflammatory cytokines, microbial metabolites, and neuroactive compounds that modulate neuronal activity, blood-brain barrier integrity, and microglial function. These alterations may promote neuroinflammation, impaired synaptic plasticity, and dysregulated emotional and cognitive processing, thereby contributing to the onset and progression of psychiatric, neurodegenerative, and neurodevelopmental disorders.

Various lines of evidence suggest that the gut-associated immune system can influence the nervous system, and it was noted in various neurological disorders, neurodegenerative diseases, and psychological illnesses. Toll-like receptors (TLRs), which are involved in the activation of various immune cells, play a major role in maintaining gut microbe-associated homeostasis of the immune system (Rogier et al., 2015). This immune interaction is mediated through the recognition of microbial-associated molecular patterns (MAMPs) by host pattern-recognition receptors. Among the most well-characterized microbial components are lipopolysaccharides (LPS) derived from Gram-negative bacteria, and peptidoglycans (PGN) and lipoteichoic acids (LTA) derived from Gram-positive bacteria. These microbial molecules are recognized by specific TLRs, particularly TLR4, which is the principal receptor for LPS, and TLR2, and are widely implicated in the recognition of PGN, LTA, and other bacterial cell wall-associated ligands (Rhee, 2011; Rhee, 2014; Zamyatina & Heine, 2020). Upon ligand binding, these receptors activate downstream intracellular signaling cascades involving adaptor molecules such as MyD88 and TIRAP, leading to the activation of NF-κB and MAPK pathways and the subsequent release of pro-inflammatory cytokines, including IL-1β, IL-6, and TNF-α (Rhee, 2011). These cytokines can compromise intestinal barrier integrity, promote systemic inflammation, and influence central nervous system function, thereby contributing to gut–brain axis dysregulation and neuroinflammatory processes associated with psychiatric disorders. These activated gut-associated immune cells produce proinflammatory cytokines, which can modulate neuronal functions upon reaching the brain (Dantzer et al., 2008). Further, cytokines are also proven to influence GB signalling (Sampson and Mazmanian, 2015). Various studies illustrated that any change in the gut microbiota in early life can increase the risk of developing immune disorders later (**Figure 2**) (Penders et al., 2007; Sherman et al., 2015).

Microbiome-induced psychiatric effects cannot necessarily be attributed to microbial taxa per se, but rather to the dynamic interplay between microbial activity and host biology. It has been suggested that host genetics, immune status, hormone sensitivity, dietary factors, drug use, and initial metabolic condition may play a role in determining the effect that microbially derived signals have on the host organism. This would imply that, even in the case of identical microbial taxa or manipulations, the resulting neurobehavioral consequences may differ from individual to individual. In light of these insights, the interaction between the microbiome, gut, and brain ought to be considered a co-regulation process involving the host and microbiome, whereby psychiatric vulnerability and treatment outcome arise from the action of both microbial products and host susceptibility.

## 3 Psychobiotics: definition and mechanisms of action

**Figure 3:**
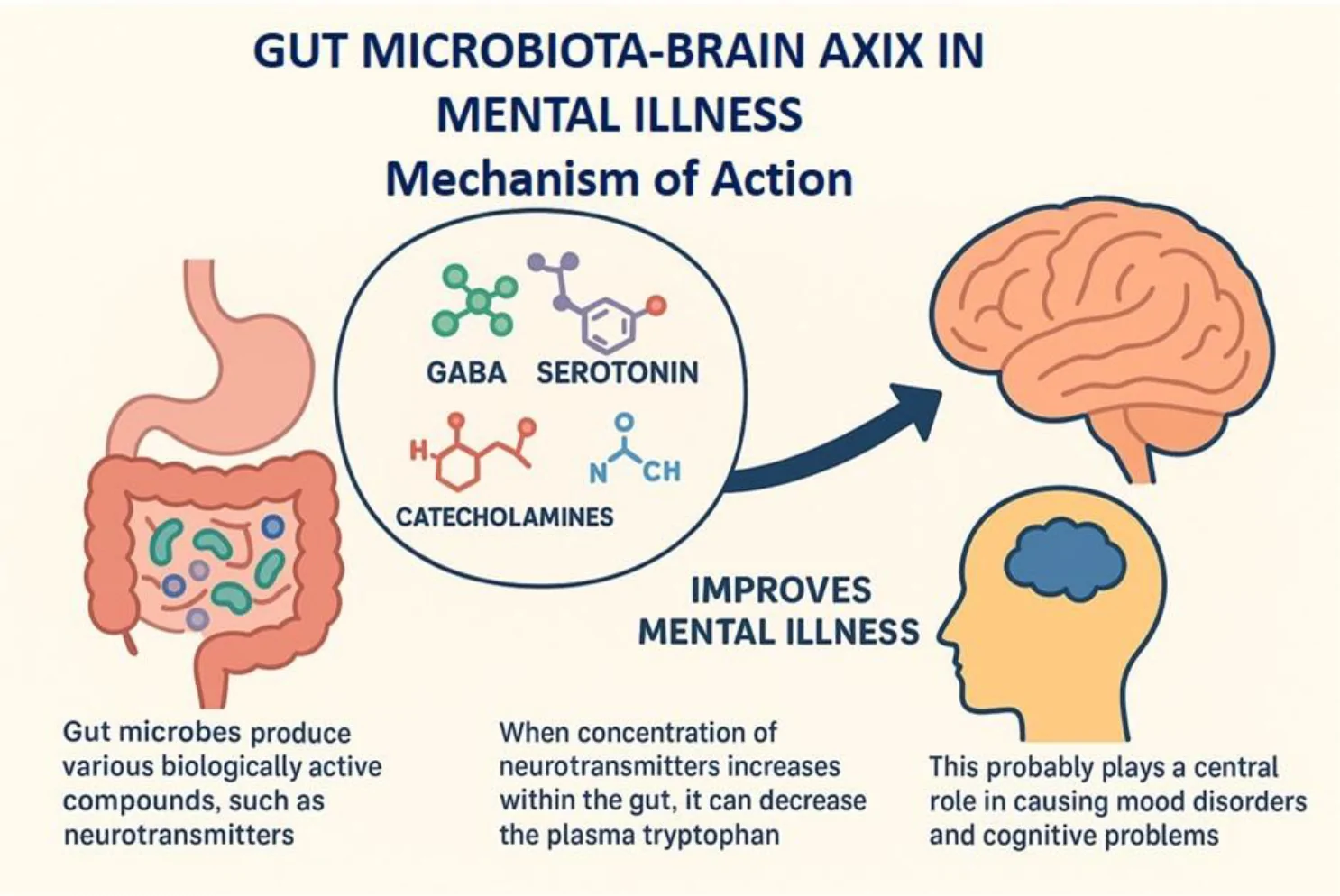
Proposed mechanism s by which the gut microb iota-brain axis contributes to mental illness. This schematic summarizes the pathways through which gut microbiota may influence central nervous system function and behavior. Gut microbes are capable of producing or modulating neuroactive molecules, including gamma-aminobutyric acid (GABA), serotonin, and catecholamines, which can affect neurotran smission, synaptic activity, emotional regulation, and cognitive performance. In addition, these microb ial signals may interact with immune, endocrine, and neural pathways to alter stress responses, mood regulation, and higher-order brain functions, thereby contributing to the pathophysiology of mood disorders and cognitive dysfunction.

Psychobiotics are living organisms, prebiotics, or synbiotics that exhibit beneficial effect in patients with psychiatric illness when administered in sufficient concentration (Dinan et al., 2013). Probiotics such as *Lactobacillus* sp, *Bifidobacterium* sp have several health benefits and are commonly used to treat any gut abnormalities. Some of these microbes (*Lactobacillus acidophilus, L. casei, Bifidobacterium infantis*, etc.), are also known to produce neurotransmitters, like serotonin, norepinephrine, etc. These microbes act on neurochemical receptor expression through GB axis, thereby causing psychotropic effects (antidepressant and anxiolytic) (Barrett et al., 2012; Akkasheh et al., 2016; Selhub et al., 2014). Furthermore, increased concentrations of neurologically active molecules such as gamma-aminobutyric acid (GABA), serotonin, and catecholamines may alter plasma tryptophan levels, which can subsequently influence brain function and modulate psychiatric conditions (**Figure 3**) (Akkasheh et al., 2016).

Significantly, emerging trends in the study of psychobiotics in the recent years demonstrate that the ability to serve as therapeutic agents does not depend only on the taxonomy of microorganisms, but also their biological functionality encompasses modulation of neurotransmitter precursors, SCFA production, epithelial barrier maintenance, immune regulation, and HPA axis interactions — pathways that are examined in mechanistic detail in the sections below. Against this backdrop, contemporary literature has found that psychobiotics can be better understood in terms of functional specificity at the strain or consortium levels rather than simply at the genus level alone (Rahmannia et al., 2024; Ķimse et al., 2024). Similarly, emerging research data further confirm the hypothesis that combinational therapy using several strains might be associated with enhanced bio-behavioral outcomes due to synergistic action within multiple pathways, such as inflammation, HPA axis, and neuroactive substances synthesis. This information adds to the growing concept regarding the need for viewing such psychobiotic strategies as functionally specific manipulations of gut microbiota rather than simple probiotic therapy. Nonetheless, despite the significant potential of psychobiotic therapy, its application is constrained by methodological inconsistencies, low numbers of subjects involved in psychiatric studies, and the lack of long-term human studies addressing specific disorders (Meng et al., 2025). Moreover, it cannot be commonly stated that such processes operate in a uniform manner among all possible candidates for use as psychobiotics. Such findings have led scientists to abandon the idea that all positive probiotics may also be regarded as psychobiotics and to adopt a more refined approach (Rahmannia et al., 2024; Gholian et al., 2024; Śliwka et al., 2025).

## 4 Role of GB axis in mental illness

The bidirectional communication of the gut and brain can influence the human health through five proposed routes. Although the mechanism of action of GB axis has not yet been fully explored, initial evidences suggest that the gut microbiota or psychobiotics can produce hormones, metabolites or immune factors, which act on cognitive development. Even though the neuronal, immunological, hormonal, and metabolic pathways can be viewed as distinct entities to facilitate understanding, they function as a highly integrated network in the MGB axis. Each of these routes is examined in the following subsections. Importantly, rather than treating these subsections as independent biological routes, the mechanistic content below should be read as a unified systems-level framework — linking microbial products, host receptor binding, intracellular signaling cascades, and functional neuroimmune and neuroendocrine outcomes.

### 4.1 Mechanistic perspective on MGB axis

Recent evidence indicates that theMGB axis should not be viewed as a mere aggregation of discrete neural, immune, endocrine, or metabolic pathways; instead, it should be recognized as an integrated systems-level communication network that connects microbial activity to host neurobiology. Modern models characterize the MGB axis as a hierarchical and sequential mechanism wherein microbial products engage with host receptors, initiate intracellular signaling cascades, and ultimately affect neurophysiological and behavioral outcomes (Morais et al., 2021). Bautista and López-Cortés (2026) also came up with the idea of “host–microbiome decoupling, “ which stresses that problems with how the host and microbes work together could lead to changes in the immune, endocrine, metabolic, and neuropsychiatric systems that are not normal.

In this integrated framework, the mechanistic sequence can be envisioned as four interrelated stages:

1. signaling molecules made by microbes,
2. recognition of host receptors,
3. activation of intracellular signaling,
4. functional effects on the brain and behavior that happen later.

Instead of utilizing a process based only on individual biological pathways, the relationship between the microbiome and psychological wellbeing takes place in a sequence of events, whereby microbial components react with their corresponding receptors. This creates a cascade of signaling pathways that change the physiological conditions, such as the state of the barrier function, inflammation, neuroendocrine system, neurotransmitters, and behaviour itself.

In the mechanistic sequence, the initial phase of microbiome–host communication encompasses microbial metabolites and structural products produced within the intestinal milieu. These substances consist of LPS, PGN, SCFAs such as butyrate, acetate, and propionate, tryptophan-derived metabolites including indoles and kynurenines, bile acid derivatives, lactate, and microbially produced neurotransmitter precursors (Needham et al., 2020; Verma et al., 2024; Zhou et al., 2025). These microbial compounds function as biologically active signaling intermediates that can affect various physiological functions. Dysbiosis-related changes in microbial metabolite production may play a role in neuropsychiatric symptoms, including stress vulnerability, neuroinflammation, cognitive impairment, anhedonia, and mood disorders, irrespective of the specific bacterial taxa implicated (Śliwka et al., 2025).

Further, the bioactive products of microbes influence their target host physiological functions via interactions with receptor proteins present on various cells within the intestine, including enterocytes, enteroendocrine cells, immune cells, vagal afferents, and other peripheral tissues (Rhee, 2011; Zamyatina & Heine, 2020; Walsh and Zemper, 2019). Some examples of these receptors include pattern-recognition receptors like TLRs (TLR2 and TLR4), metabolite-sensing G-protein coupled receptors (GPR41 and GPR43), and aryl hydrocarbon receptors (AhR) that mediate transcriptional and immune signaling.

Receptor binding of microbial ligands facilitates the bidirectional communication between the gut microbiome and host systems. SCFAs that bind to GPR41/GPR43 receptors might modulate enteroendocrine cell secretion and vagal nerve function. Conversely, LPS-induced TLR4 receptor activation may promote inflammatory responses in conditions linked to neuroimmune dysfunction. The activation of AhR signaling pathways by tryptophan metabolites might influence mucosal immunity, neuroinflammation, and transcriptional regulation.

Once the receptors are stimulated, various intracellular signaling pathways are activated, allowing microbial information to be incorporated into host cellular responses. These pathways include MYD88-dependent signaling, nuclear factor kappa B (NF-κB), mitogen-activated protein kinase (MAPK) pathways, NLRP3 inflammasome activation, and histone deacetylase inhibition via SCFAs (Yang et al., 2023).Signaling pathways represent major molecular junctions that connect microbial stimuli with immune response activation, epithelial barrier control, stress responses, and neuronal signaling. The stimulation of NF-κB and MAPK pathways triggers the secretion of cytokines and inflammatory signaling molecules, while NLRP3 inflammasome activation facilitates neuroimmune disruption and neuroinflammation. Moreover, butyrate-induced histone deacetylase inhibition can have neuroprotective and anti-inflammatory effects through epigenetics.

Recent research has also underscored the role of transcriptional and epigenetic regulation in the effects of the microbiome on mental health. The products generated by gut bacteria ‘ might regulate gene expression through chromatin remodeling, histone modifications, and receptor-mediated gene regulation that affect the expression of genes involved in stress response, neuroplasticity, inflammation, and synaptic function (Verma et al., 2024; Lee et al., 2025).

The functional consequences of the above signaling processes affect numerous physiological aspects. Specifically, the processes discussed above impact permeability of the intestine, immune response, the function of the vagus nerve, the activity of HPA axis, and neurotransmitter metabolism associated with serotonin, dopamine, GABA, and catecholamines (Cryan and Dinan, 2012; Sampson and Mazmanian, 2015; Lee et al., 2025).

In case of the neuroimmune and neuroendocrine systems, neurotransmission, which is an important aspect of these processes, is conducted in two ways. These can occur via direct and indirect pathways using enteric nervous system, vagus nerves, microbes’ neurotransmitters and enteroendocrine cells (Schmidt, 2015). Changes in tryptophan metabolism and decreased tryptophan concentrations can further impact serotonergic processes that have been associated with psychiatric disorders (Akkasheh et al., 2016).

**Figure 4:**
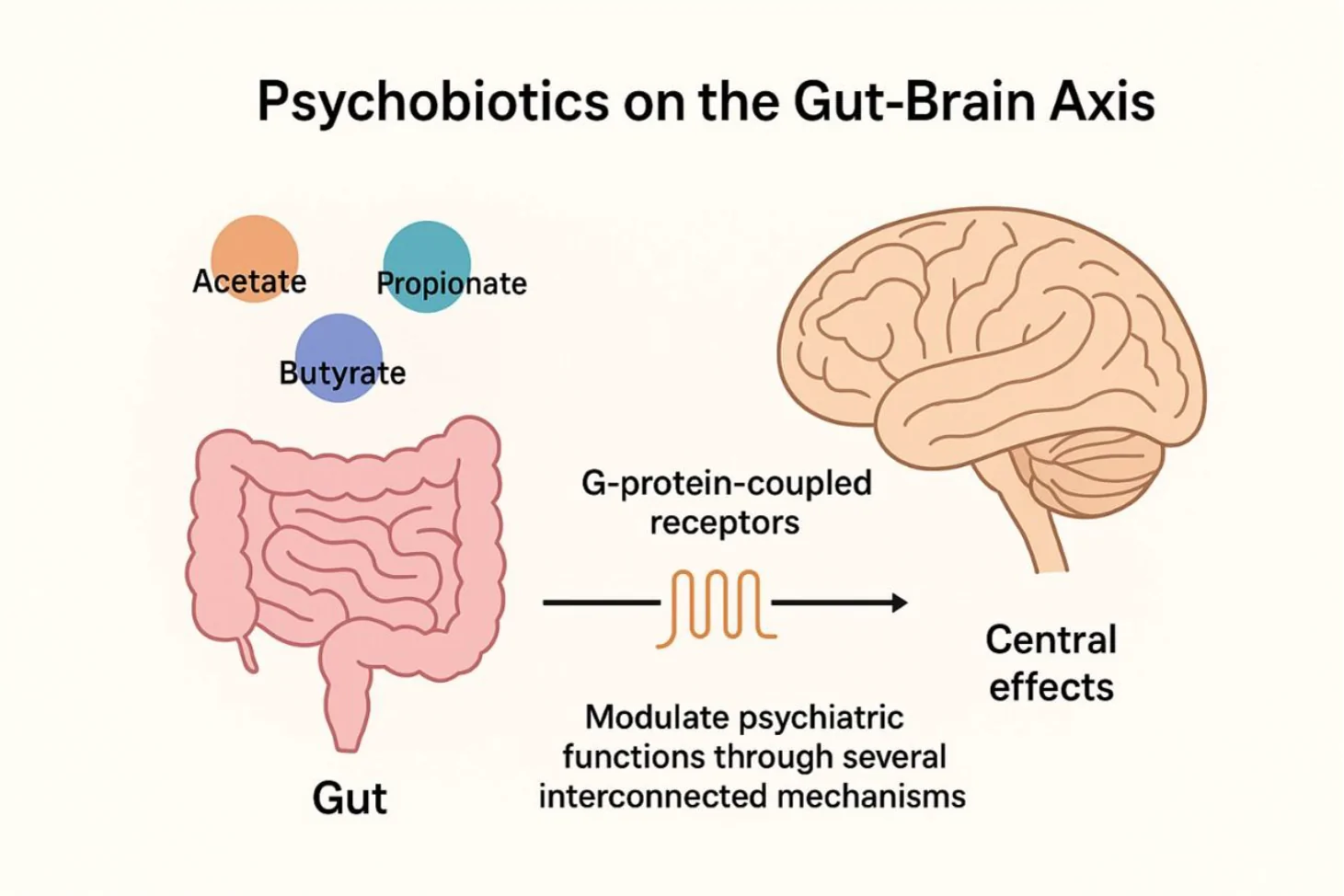
Proposed mechanisms by which gut microbiota-derived short-chain fatty acids influence brain function and psychiatric disorders. This schematic summarizes the role of short-chain fatty acids (SCFAs), major metabolites generated by gut microbial fermentation, in the regulation of the gut-brain axis. SCFAs may modulate central nervous system function through multiple pathways, including immune signaling, neuroinflammatory responses, neurotransmitter regulation, blood-brain barrier integrity, and epigenetic modulation. By influencing these interconnected processes, SCFAs may alter mood, cognition, stress responsiveness, and other psychiatric-related functions, thereby contributing to the development or progression of mental illness.

Further, SCFA and other microbial metabolites act both as signaling mediators and as regulators of neuronal plasticity, glial activation, and immune modulation at the metabolic level (**Figure 4**) (Stilling et al., 2014). Meanwhile, disruption of the HPA axis may result in prolonged stress responses, hormonal dysfunction, mood alterations, and impaired cognition.

Increasingly, it has become clear that immune-inflammation aspects of the MGB axis constitute part of a barrier-immune-brain signaling sequence rather than separate episodes of inflammation. Dysbiosis may lead to impairment of tight junctions between epithelial cells and changes in the structure of the mucus layer, exposing the body to microbial products and triggering innate immunity pathways, including TLR signaling and activation of NLRP3 inflammasome. This cascade leads to disruption of the BBB, microglia activation, neuroinflammation through activation of astrocytes, and altered neurotransmitters’ synthesis (Montiel-Castro et al., 2013; Foster and McVey Neufeld, 2013; Yang et al., 2023).

Overall, these results confirm that the role of psychobiotics and FMT can be understood via different biological pathways working together as opposed to one specific pathway. As such, the MGB axis should be considered a complex biological system that involves various levels of communication between the host and microorganisms. These interactions include, but not limited to, metabolite production, receptor interactions, immune modulation, neuroendocrine regulation, neuronal circuits, and epigenetic programming to affect mental well-being and diseases (Binda et al., 2024; Lee et al., 2025).

## 5 Psychobiotic evidences in preclinical and clinical studies

According to previous studies, psychobiotics may affect emotionality, stress response, neuroimmunological signaling, and cognition through gut-brain microbiome communication. But the existing data show large discrepancies between results of studies in preclinical research and in humans. It should be noted that the focus on studying psychobiotic effects has shifted from whether they have any neuropsychiatric impact to how these effects happen in various biological and experimental conditions. The evidence in this section is organized thematically around three key areas: (1) strain-specific effects and mechanistic data from preclinical research; (2) variability across clinical studies, including differences between healthy subjects and those with psychiatric diagnoses; and (3) methodological limitations that currently constrain translational interpretation. Preclinical and clinical findings are presented in separate subsections to facilitate clearer interpretation of the strength of evidence at each level.

### 5.1 Specific microbial strains and mechanistic evidences from preclinical research [preclinical evidence]

**Figure 5:**
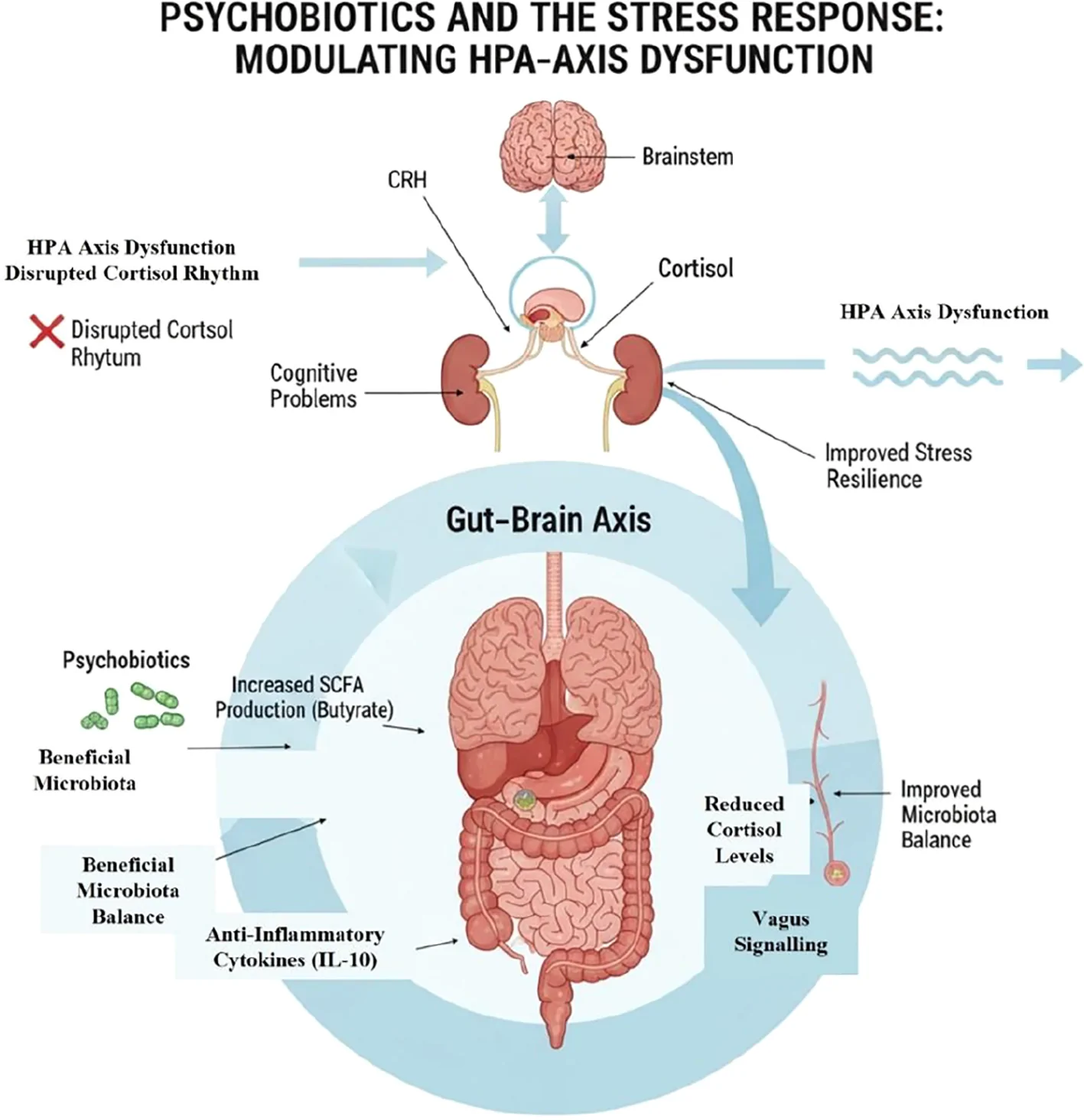
Proposed mechanisms by which psychobiotics modulate hypothalamic-pituitary-adrenal axis dysfunction and stress responses through the gut-brain axis. This schematic summarizes the potential effects of psychobiotics on hypothalamic-pituitary-adrenal (HPA) axis regulation and stress-related neurobiological pathways. By influencing gut microbial balance, inflammatory signaling, neurotransmitter production, and neuroendocrine communication, psychobiotics may help normalize stress-induced physiological responses. These actions may contribute to reduced cortisol secretion, improved HPA axis homeostasis, and enhanced stress resilience, thereby supporting emotional regulation and mental health.

The most compelling mechanisms explaining the neuropsychological actions of probiotics are those revealed in preclinical animal research. It provides a clear evidence that certain bacterial strains can affect neurotransmission, immunity, stress, and behavioral changes through vagal, HPA axis, neuroimmune signaling, and microbial metabolic pathways (**Figure 5**).

The discovery of the importance of strain specificity is one of the key results derived from experimental studies. Indeed, various probiotic strains can have different impacts on the neurobehavioral and biochemical functioning of mammals. This evidence indicates that psychobiotic effects cannot be observed for all types of probiotics. In this context, *L. rhamnosus* has been found to influence GABAA receptor expression in the brain of animals and decrease their anxiety and depressive symptoms via a vagus nerve-dependent pathway. At the same time, in animals subjected to vagotomy, these probiotic effects were not observed (Bravo et al., 2011).

Further evidences support the strain-specificity of functional effects. *L. helveticus* showed anxiolytic and memory-preserving effects under stress-induced conditions (Ohland et al., 2013). In contrast, *B. longum* NCC3001 had anti-anxiety effects in colitis-induced anxiety mediated by the vagal pathway (Bercik et al., 2011). Combinations of strains have also been proven effective, as seen in the improvement of cognitive function in diabetic animals and depression symptoms following myocardial infarction (Davari et al., 2013; Bercik et al., 2013). Chronic administration of *B. longum* 1714 and *B. breve* 1205 produced anti-anxiety behaviors, whereas *L. helveticus* NS8 displayed antidepressant-like effects via modification of gut-brain communication channels (Savignac et al., 2014; Liang et al., 2015).

In summary, it can ne noted that these studies confirm the role of the microbial strain’s characteristics, metabolic activities, and host environment in influencing the effects of probiotics on brain health. Based on pre-clinical evidence, vagal pathways, serotonergic and dopaminergic systems, immune regulation, SCFAs, and HPA axis normalization were all implicated in these effects (Ait-Belgnaoui et al., 2012).

### 5.2 Human clinical trials: small benefits with large variability [clinical evidence]

Unlike in animal experiments where results were relatively uniform, studies conducted on humans reveal considerable heterogeneity of results. Randomized controlled trials with both healthy people and those suffering from depression, anxiety, or other conditions associated with elevated stress levels usually report only mild benefits. These benefits, which include improved mood, reduced feelings of stress, and enhanced emotional functioning, can be small and rarely consistent (Messaoudi et al., 2011; Akkasheh et al., 2016).

Another trend in clinical research is the large inter-study variability. Variations in probiotics’ strains used in the experiment, dosage, period of treatment, type of preparation, initial state of microbiota of participants, as well as personal characteristics and methods of measurement result in contradictory conclusions (Rahmannia et al., 2024; Mosquera et al., 2024). Thus, it is particularly important to note that positive results were obtained predominantly with respect to subjects with existing psychological problems. Moreover, many clinical trials utilize different rating scales and diagnostic criteria for the measurement of psychological endpoints, making it difficult to compare the findings directly among the different researches. Further, dietary habits, drug consumption, and variations in lifestyle and microbiota among others may also affect interpretation of the results obtained from the human trials.

### 5.3 Factors causing variability in psychobiotics studies

From the preclinical and clinical perspectives, it was noted that certain factors consistently play a role in affecting the outcome of psychobiotic studies. The major factors include (i) Strain-Specific Functional Variability, (ii) CFU, Viability, Duration of Administration, and Single/Multi-strain Formulations, (iii) Host-Dependent Effects, (iv) Experimental Design Differences.

A very potent and reproducible source of variability is strain-specificity, since even genetically similar bacteria may greatly vary in terms of their ability to generate neurotransmitter precursors, SCFAs, immunomodulatory metabolites, and/or vagal signaling. This means that results obtained for a single probiotic strain cannot be directly applied to other strains from the same genus or species.

Further, the variability might also be influenced by the dose (CFU) or survival of administered bacteria, the total period of the intervention, as well as by whether probiotics were administered alone or in combination with other strains. Some authors indicate the necessity of long-term treatments in order to observe neuropsychiatric effects, while short courses might have an impact on transient stress.

In addition, the individual characteristics of the host might also play a major role in clinical variability observed between participants of different clinical trials. For example, subjects diagnosed with depression, chronic stress, inflammation, gastro-intestinal problems, or metabolic dysfunction are more (Cani et al., 2012) likely to be affected by probiotics compared to healthy people. Finally, a high degree of variability can be found in the design of psychobiotic experiments. Behavioral tests, psychological assessment methods, time of follow-up, control of diet, and statistical analyses represent some of the parameters that contribute to the lack of consistency and reproducibility of results, making meta-analyses problematic. Preclinical studies differ in their stress models, genetic backgrounds of animals used, living conditions, and techniques of microbiota modification, potentially affecting the resulting behavior.

### 5.4 Translation from preclinical to clinical evidence

The translation of the mechanisms established using preclinical models to humans remains a significant problem in psychobiotic research. *In vivo* experiments allow an experimental manipulation of the gut microbiota, environment, diet, and stress, facilitating more accurate analysis of the mechanisms involved. In humans, however, many confounders exist, such as different diets, medications, genetics, lifestyles, environment, and even microbial differences between individuals. Accordingly, findings obtained from animal models fail to consistently correlate with clinically relevant outcomes in patients with mental disorders. Although the available evidence clearly demonstrates that there is a two-way relationship between the gut microbiota and the brain, the exact strength and clinical significance of such associations in psychiatric conditions are yet to be elucidated (Mayer et al., 2014).

Given the current understanding of the relationship between psychobiotics and mental health disorders, it can be argued that the use of psychobiotics for clinical purposes is still premature, as their efficacy in treating or alleviating psychiatric symptoms is not fully understood till date. Instead, psychobiotics represent a biological mechanism by which MGB relationships can be modulated, depending on numerous variables, including strain specificity.

### 5.5 Human evidence for psychotropic actions of probiotics

The available evidences on the effects on humans indicates a number of convergent trends, notwithstanding its limited scope and consistency. *L. helveticus* and *B. longum* supplementation decreased psychological discomfort in a randomized controlled experiment (Messaoudi et al., 2011), and fermented milk consumption caused quantifiable changes in emotional brain responses in healthy volunteers after four weeks (Tillisch et al., 2013). Fermented dairy products have also been linked to benefits in mood and emotional state, and multi-strain formulations have shown decreased cognitive reactivity to sorrow and violent thoughts (Barrett et al., 2012; Benton et al., 2007). Although the populations under study are diverse and the effect sizes are often small, these results are consistent in direction. Furthermore, probiotic consumption was linked to better subjective well-being in an occupational setting (Mohammadi et al., 2015, 2016) and reduced physical stress symptoms in university students during academic exam times (Kato-Kataoka et al., 2016).

Children with autism spectrum disorder have been found to exhibit distinctive patterns of microbial dysbiosis (Darwesh et al., 2024), and there is preliminary evidence that probiotic supplementation may promote epithelial integrity, lower inflammation, and modify ASD behavioral characteristics (Critchfield et al., 2011). More specifically, hepatic encephalopathy, depression, ASD, and disease-related anxiety have all been the subject of clinical trials that have yielded suggestive signals of benefit (Zhou and Foster, 2015); however, none of these results are yet conclusive. Although the results available are not adequate to design therapeutically useful biomarkers, but emerging work on precision psychobiotics remains conceptually attractive (Darwesh et al., 2024). When considered collectively, the human evidence does not yet prove efficacy, but it establishes that psychobiotic therapies have directionally consistent, biologically plausible effects on mood, stress, and cognition that call for more extensive and methodologically rigorous trials.

There are various limitations on the existing evidence. One of the primary drawback is the translational gap between established preclinical evidence and inadequate human evidence. Although animal experiments have provided important mechanistic insights — as reviewed in the preceding sections — their direct correlation with human psychiatric conditions remains uncertain. This can be attributed by the fact that human research is limited due to sample size limitations, limited duration of treatment, baseline psychological state variations etc. Moreover, substantial variations across occur in studies that includes, types of probiotics used (strain & combination), dosage (CFUs), length of treatment period, and populations and preparations used. Further, variations in psychological measures and biomarkers can also cause diversification in outcomes.

### 5.6 Critical synthesis of existing psychobiotic evidence

Collectively, the psychobiotic literature provides biological plausibility to a possible association between selective microbial treatments and mental health outcomes. However, the quality of evidence across different studies remains highly variable. According to recent systematic reviews and meta-analyses, it can be argued that the administration of psychobiotics might potentially have positive effects on the symptoms of depression, anxiety, and stress. However, there exists significant variability regarding both the magnitude and consistency of the reported results (Rahmannia et al., 2024; Peng et al., 2024; Mosquera et al., 2024; Śliwka et al., 2025). More importantly, recent works suggest that any such effects are highly dependent on the probiotic strain used for the respective research and significant discrepancies can occur from differences in dosage and treatment protocol, host microbiome composition, and type of psychological assessments (Rahmannia et al., 2024; Gholian et al., 2024; Śliwka et al., 2025). Furthermore, it is essential to note that most studies conducted among humans involve fairly limited sample sizes and short interventions, as well as lack proper diagnosis for various psychiatric conditions (Mosquera et al., 2024; Ķimse et al., 2024).

## 6 Fecal microbiota transplantation in mental health

### 6.1 Fecal microbiota transplant

**Figure 6:**
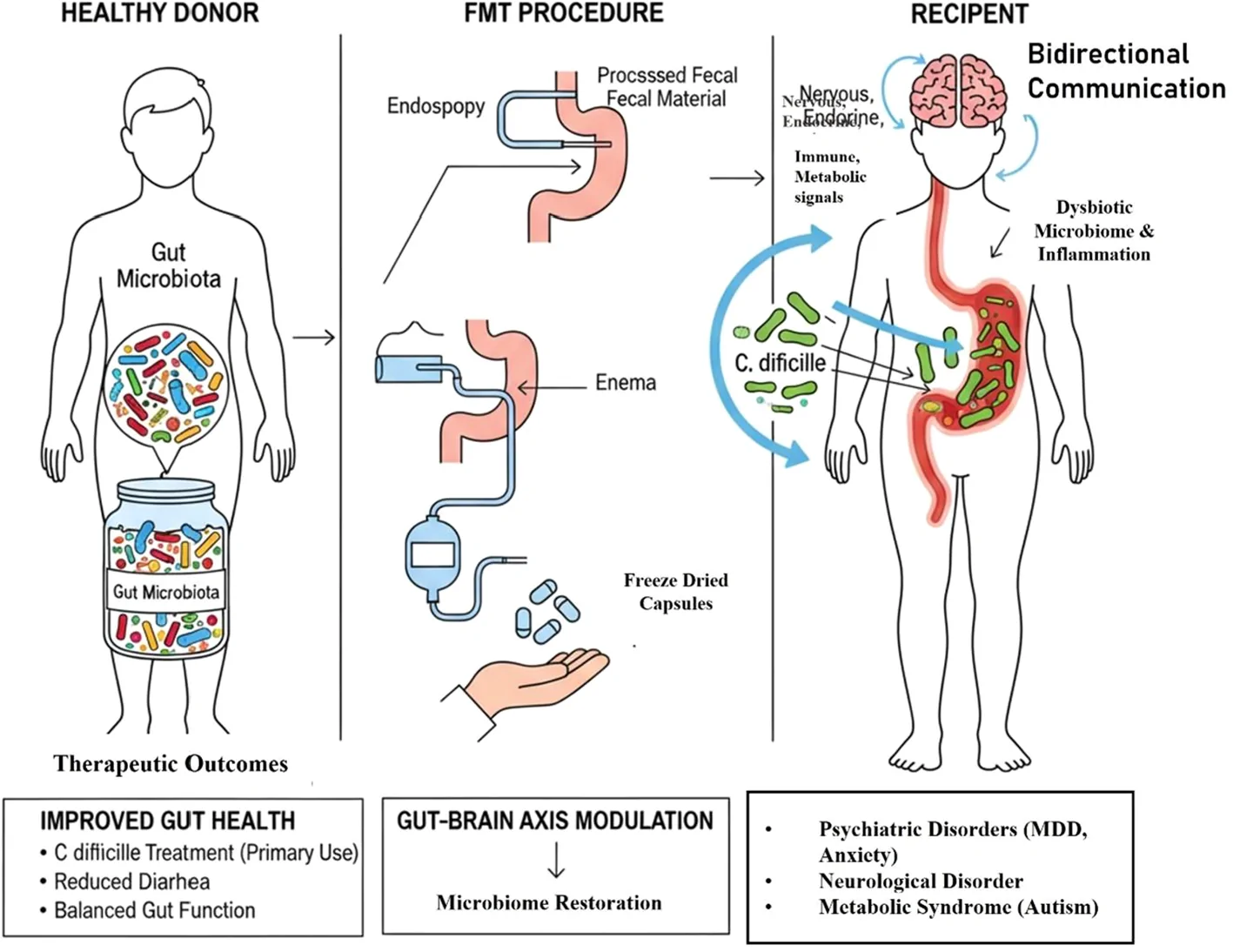
Proposed mechanism sby which fecal microbiota transplantation modulates the gut-brain axis and supports therapeutic outcomes. This schematic summarizes the process of fecal microbiota transplantation (FMT) and its potential role in re-estab lishing a healthy gut microbial ecosystem and restoring gut-brain axis signaling. Through modulation of microbial composition and function, FMT may influence intestinal barrier integrity, immune and inflammatory responses, microb ial metabolite production, and neuroendocrine and neural communication pathways. These interconnected effects may contribute to improved gastrointestinal and systemic homeostasis, with potential therapeutic implications for psychiatric, neurological, and metabolic disorders.

A variety of methods have been employed to investigate the relationship between the gut microbiome and the GB axis. These include altering the microbial composition through the administration of probiotics and antibiotics, the use of germ-free animal models, and most notably FMT (Zhang et al., 2012). The clinical approval for FMT is focused at combating *Clostridium difficile* infections (CDI). This infection is common in elderly patients after continuous antibiotic treatment, which disrupts the gut’s microbial balance. In FMT, the beneficial microorganisms are transplanted from a donor to the recipient, to restore diverse gut ecosystem. This treatment, involves methods like colonoscopy, enemas, or oral capsules containing dehydrated fecal material (de Groot et al., 2017b) (**Figure 6**).

### 6.2 FMT in the context of psychiatric illness

MDD and anxiety disorders are two of the most common mental health disorders. Persistent sadness or a substantial diminution of interest or pleasure in life, typically combined with a variety of emotional and somatic symptoms, characterizeMDD (American Psychiatric Association, 2013). Further, a family of related illnesses known as anxiety disorders feature these symptoms at an extreme, debilitating level. Considering their prevalence, it is important to prioritize them while considering FMT in the context of psychiatric disorders.

Although there are pharmacological treatments available for both disorders, many patients either reject them due to adverse side-effects, stigmatization, or do not sufficiently respond to traditional treatments. With these hurdles, alternative approaches centered around the gut–such as FMT–are surfacing as potential strategies to circumvent therapeutic barriers and address mood and anxiety disorders. With evidence from ongoing research on GB axis, researchers hypothesize that restoring balance to the gut microbiome by FMT from healthy donors can alleviate psychiatric symptoms.

Although research in FMT in psychiatric disorders has been sparse, advances in related neurological and neurobehavioral disorders are providing important cross-validation for approaches targeting the microbiome for therapeutic intervention. For example, a recent study from a randomized phase 2 clinical trial has shown that repeated donor FMT has therapeutic benefits for both motor and gastrointestinal symptoms in drug-naive Parkinson’s disease. These results provide further importance to the therapeutic value of microbiota modulation in brain disorders. Although Parkinson’s disease and psychiatric disorders are different in etiology, both involve brain disorders, and such research has value in considering FMT in a broader therapeutic context (Zhang et al., 2026). However, since this field is still emerging, there are relatively fewer human studies investigating the therapeutic potential of FMT in psychiatric conditions. Importantly, the current evidence for FMT in psychiatric populations remains limited with respect to both efficacy and safety. Most available data are derived from preclinical studies, while human investigations remain few and are often constrained by small sample sizes, short follow-up periods, heterogeneity in donor selection, route of administration, treatment schedules, and psychiatric outcome measures. As a result, the therapeutic efficacy of FMT for conditions such as MDD and anxiety cannot yet be considered established. Further, safety also remains an important concern, particularly regarding long-term tolerability, donor screening, microbial engraftment, unintended transfer of undesirable microbial traits, immune-related effects, and interactions with ongoing psychotropic medications. Therefore, FMT in psychiatry should currently be regarded as experimental and investigational rather than clinically established.

Although the use of FMT in psychiatry remains experimental and is not approved by regulatory authorities, it is progressing towards more structured clinical evaluation. Several ongoing clinical trials are currently exploring the role of FMT in psychiatric and related neurobehavioral conditions. For example, ongoing studies are assessing adjunctive FMT inMDD and repeated oral FMT interventions in disorders such as schizophrenia, with the aim of evaluating both clinical efficacy and safety outcomes. These trials are expected to provide more robust evidence regarding treatment response, tolerability, and microbiome-related mechanistic changes in psychiatric populations.

In contrast to psychobiotics, there is less scientific support for the use of FMT in psychiatry due to its relative novelty and heterogeneity of methods used. According to recent meta-analyses, despite some promises observed in improving depression and anxiety symptomology through the use of FMT, most of the supporting evidence comes either from preclinical or translationally based research or very early phase clinical trials rather than properly-powered psychiatric studies (Vasiliu, 2023; Zhang et al., 2025; Fu et al., 2026). Additionally, recent methodological assessments have highlighted significant inconsistency in donor criteria, gut microbiome depletion techniques, colonization verification, modes of administration, administration regimens, and behavioral measures in the existing FMT psychiatric studies (D'Onofrio et al., 2026). Considering the available literature, this review highlights key findings from both preclinical and clinical research to explore the impact of transferring endogenous microbiota on psychiatric symptoms. Moreover, some studies have alsp explored related conditions that often accompany poor psychological well-being, such as alcohol dependence and eating disorders like anorexia.

The present level of evidence in the field of FMT in psychiatry covers different levels, each having their own advantages and disadvantages. The preclinical level gives the most consistent evidence regarding the mechanisms by which FMT influences psychopathology. This is explained by the fact that it has been proven in numerous studies that the transfer of microbiota of affected individuals leads to behavioral and neuropsychiatric alterations in experimental animals, proving the involvement of the microbiome in the development of psychiatric disorders (Kelly et al., 2016; Zheng et al., 2016). However, there is no clinical evidence regarding FMT due to the lack of studies among patients with various mental conditions.

Furthermore, there is another part of the evidence that comes from other medical conditions such as Parkinson’s disease and metabolism where FMT has shown its efficacy in relation to GI tract function, metabolism, and movement (de Groot et al., 2017a; Zhang et al., 2026). While it helps to understand how one can work with the manipulation of microflora and brain–gut connection, these findings cannot be seen as an evidence base for the treatment of mental diseases as they aid only to generate new hypotheses about potential mechanisms (D'Onofrio et al., 2026).

Summing up, it appears that while there is no doubt that FMT has great potential, it is important to mention that current evidence is dominated by preclinical and indirect results with a significant lack of well-designed studies among psychiatric patients. It implies that FMT should be perceived as an investigational intervention and not as a therapy in this field (Vasiliu, 2023; D'Onofrio et al., 2026).

### 6.3 Key targets associated with FMT in treating depression

Although the mechanisms underlying the psychiatric effects of FMT are not yet fully understood, recent studies have begun to identify specific host pathways that may mediate microbiota-driven behavioral changes. Among these, the Sigma-1 receptor (Sig-1R) and the NLRP3 inflammasome have gained attention because both are strongly linked to depression-related pathology. While Sig-1R is primarily associated with neuroplasticity and neuronal survival, the NLRP3 inflammasome reflects the inflammatory and immune dimension of the gut–brain axis. Together, these pathways provide complementary insight into how altered gut microbiota may contribute to depressive phenotypes and how FMT may exert therapeutic effects. Among recently emphasized immune pathways linking the microbiota to psychiatric symptoms, the NLRP3 inflammasome has emerged as a particularly important mediator GB inflammatory signaling, neuroimmune activation, and stress-related behavioral dysfunction (Yang et al., 2023).

Sigma receptors are categorized into two types: Sigma-1 and Sigma-2 (Marcotti et al., 2023). The Sigma-1 receptor (Sig-1R) is a protein weighing about 28 kDa that functions as a molecular chaperone. It plays an important role in managing various cell activities, such as controlling calcium levels inside cells, regulating cell death (apoptosis), and maintaining the permeability of cell membranes (Vavers et al., 2023). Because of its role in maintaining the health and functionality of neurons, Sig-1R has attracted a lot of attention as a possible target for treating disorders of the CNS (Ren et al., 2022).

In one study, Li and colleagues found that mice with the Sig-1R gene knocked out exhibited depressive-like behaviors and altered gut microbiota (Li et al., 2023). It is interesting to note that these depressive behaviors improved once antibiotics were used to lower the gut bacteria in the mice. Healthy mice that were transplanted with fecal microbiota from these Sig-1R KO also displayed depression-like syndrome. Concomitant with this, there was an evident reduction in the diversity and abundance of specific gut bacteria including *Alistipes*, *Alloprevotella*, and *Lleibacterium*. This is associated with the repression of crucial signalling systems, specifically cAMP/CREB/BDNF pathway, and also showed a downward trend in neurotrophic factors such as TGF-α, NGF and CTNF.

In addition to neurotrophic and neuronal signaling pathways such as Sig-1R, inflammatory signaling pathways have also emerged as critical mediators of microbiota-associated depression. Among these, the NLRP3 inflammasome has attracted particular interest because of its central role in neuroinflammation and GB immune communication. NLRP3 inflammasome regulates inflammatory and immune reactions within GB axis. Neurons, astrocytes, and microglia in brain possess the NLRP3 inflammasome with several protein domains initiating a cascade of signals to restore cellular homeostasis (Xia et al., 2023).

Knowing how NLRP3 works and what goes wrong when it does not is crucial for depression studies due to its significant function in gut-brain-immune interaction. Zhang et al. (2025) studied chronically stressed mice who received feces transplants from NLRP3 knockout mice (Zhang et al., 2019). After this treatment, the stressed mice exhibited enhanced astrocyte function as well as reduced depressive-like behaviors. In addition, the therapy inhibited circHIPK2 elevation induced by stress, a circular RNA. These findings indicate that gut microbiota of NLRP3 knockout mice may influence astrocytes through circHIPK2, exerting anti-depressant effects and potentially representing a new therapeutic approach.

**Figure 7:**
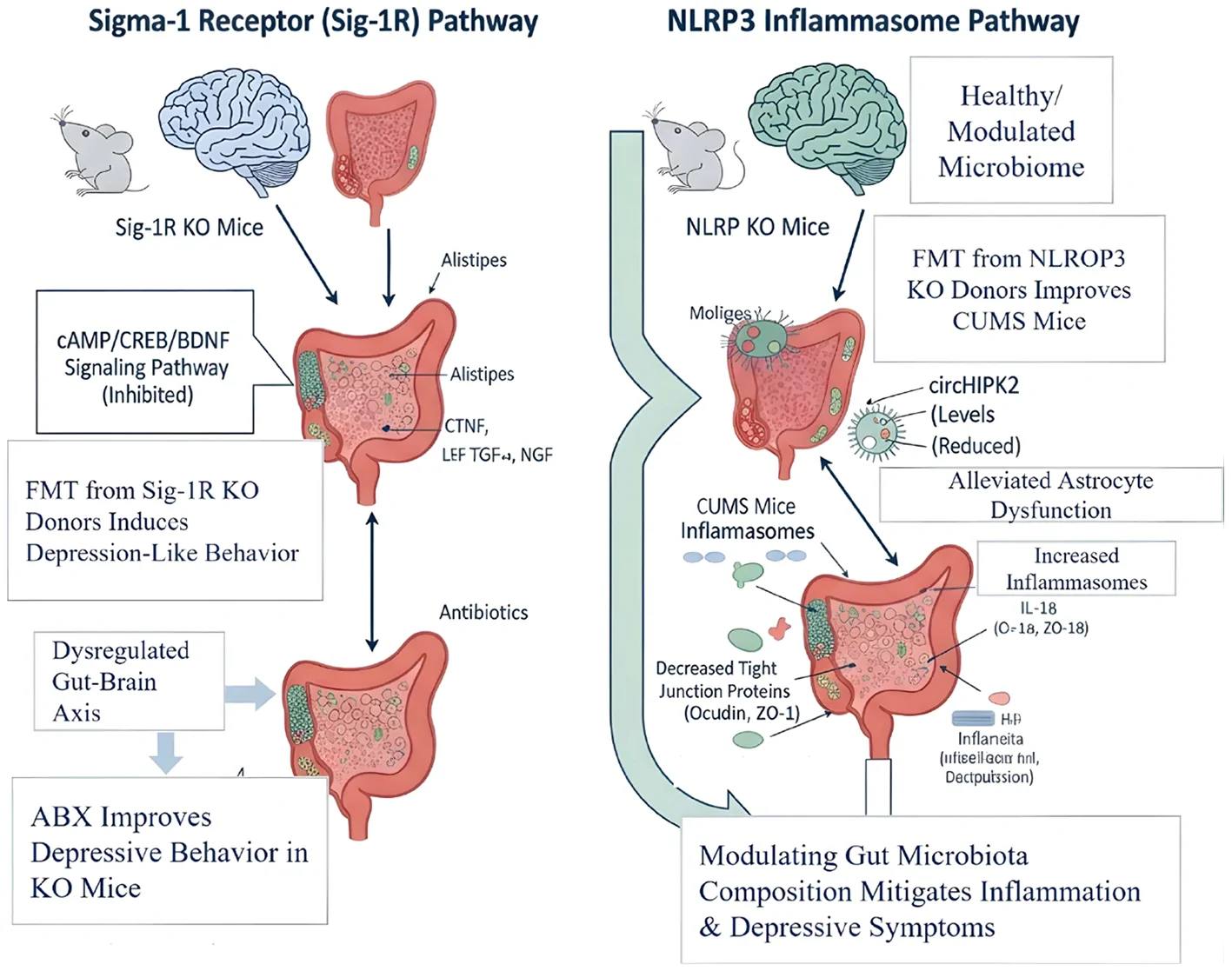
Proposed involvement of the Sigma-1 receptor and NLRP3 inflammasome pathways in the antidepressant-like effects of fecal microbiota transplantation. This schematic summarizes two key molecular targets through which fecal microbiota transplantation (FMT) may influence the gut-brain axis and alleviate depressive-like behaviour in mice. Modulation of the Sigma-1 receptor (Sig-IR) pathway may contrib ute to improved neuronal signaling, stress adaptation, and neuroprotection, whereas inhibition of the NLRP3 inflammasome pathway may reduce neuroinflammation and inflammatory cytokine production. Together, these pathways may represent important mechanistic links between gut microbial modulation and behavioural improvement in preclinical models of depression.

By giving antibiotics to rats before transplanting fecal microbiota of donors that were subjected to prolonged mild stress, Huang and co-workers explored this subject further. The recipient rats had reduced levels of gut barrier-sustaining proteins such as occludin and ZO-1, increased activity of inflammasomes, and elevated concentrations of inflammatory molecules such as IL-1β and IL-18; which are known to disrupt neurotransmitter balance, synaptic function, and stress-response systems. These changes are closely associated with core symptoms of depression, including low mood, anhedonia, fatigue, and cognitive impairments (Hassamal, 2023). With increases in some bacteria groups, including Actinobacteria and Proteobacteria, and reductions in others, including *Lachnospiraceae*, their gut microbiota community changed to become more similar to that of the stressed donors. Generally, these findings illustrate how the modulation of gut microbiota can affect NLRP3 inflammasome function, which has implications for affecting inflammation and depressive behaviors (Huang et al., 2023) (**Figure 7**).

Dysregulation of the NLRP3 inflammasome within the GB axis has important implications for psychiatric outcomes, particularly depression (McColgan et al., 2026). Heightened inflammasome activity and the resulting increase in pro-inflammatory cytokines are strongly associated with core symptoms such as low mood, anhedonia, fatigue, and cognitive impairment. Thus, microbiota-driven modulation of NLRP3 activity provides a key link between peripheral immune disturbances and the emergence and severity of psychiatric symptoms (Zhang et al., 2023).

## 7 Conclusion

The growing understanding of the MGB axis has positioned psychobiotics and FMT as promising next-generation therapeutic strategies for mental health disorders. Current evidence suggests that these interventions engage the multilayered mechanistic network described throughout this review to influence psychiatric outcomes through complementary and partially overlapping pathways. Preclinical studies provide compelling mechanistic support, while early clinical findings indicate potential benefits in conditions such as depression, anxiety, autism spectrum disorder, and stress-related disorders. Together, these observations reinforce the therapeutic relevance of microbiome-targeted approaches as potential adjuncts to conventional psychiatric care. However, the strength of evidence is not equivalent across microbiome-based interventions. Psychobiotics currently have a broader—although still methodologically heterogeneous—evidence base and greater practical feasibility for clinical translation. Whereas, FMT remains more experimental, less standardized, and supported predominantly by preclinical or indirect translational evidence. Accordingly, these interventions should not be interpreted as therapeutically equivalent, even though both are discussed under the broader umbrella of microbiome-based psychiatry.

Further, the present literature remains constrained by several important limitations. Much of the available evidence is derived from animal models, while human studies are comparatively few, often involve small sample sizes, short intervention periods, heterogeneous outcome measures, and marked variability in probiotic strains, dosages, treatment duration, and patient populations. Likewise, evidence for FMT in psychiatric indications is still at an early stage, with limited controlled clinical trials and insufficient long-term safety data. These limitations make it difficult to draw definitive conclusions regarding efficacy, reproducibility, and clinical applicability across diverse psychiatric disorders.

From a mechanistic standpoint, the research area is gradually shifting its focus away from the reductionist approaches that involve studying individual microbial strains or pathways separately. Rather, there is accumulating evidence indicating that a system-wide approach involving the role of microbial metabolites, neuroimmune interactions, epithelial barriers, gene expression in the host, and the susceptibility of the host itself determines the mental health-related effects of the microbiome.

The major limitation of the available literature is that the vast majority of the studies are preclinical, and significantly lacks large-scale, randomized, and longitudinal studies on the efficacy and safety of psychobiotics and FMT in humans. Furthermore, the significant interindividual differences in the composition of the gut microbiome, influenced by factors such as host genetics, diet, environment, and medication, make it difficult to predict outcomes in this area. The research is also affected by the fact that different studies have different methods, including sample collection, sequencing, and analysis. This variations significantly hinder reproducibility, comparison, and the development of a standard therapeutic approaches.

In addition, the scope of this review should be interpreted within its own boundaries. This manuscript primarily provides a narrative synthesis of emerging evidence on psychobiotics and FMT in mental health, rather than a systematic or quantitative meta-analytic evaluation. As such, while it highlights key mechanistic pathways and representative studies, it does not exhaustively resolve inconsistencies across study designs, microbial formulations, disease subtypes, or host-specific biological variability. This is particularly relevant given the substantial interpersonal differences in gut microbiome composition, diet, immune tone, medication exposure, and environmental factors that may strongly influence treatment response.

### 7.1 Future directions

Future research should therefore move beyond broad proof-of-concept studies towards standardized, precision-oriented microbiome therapeutics. For FMT, there is a clear need for harmonized clinical protocols covering donor screening, stool preparation, route of administration, dose frequency, and post-transplant monitoring, alongside robust safety frameworks and long-term follow-up. Similarly, psychobiotic therapies would benefit from clearer donor-independent standardization, including strain-specific characterization, mechanistic validation, and disease-targeted formulation design. Further, future studies should incorporate patient stratification frameworks to identify which subgroups are most likely to benefit from specific microbiome interventions. This may include stratification by psychiatric diagnosis, inflammatory phenotype, metabolic status, baseline microbiome composition, medication history, or symptom clusters.

A major future direction lies in the integration of multi-omics and precision medicine approaches. For example, metagenomic profiling could be used to identify microbial deficits or functional pathway disruptions in individual patients and guide the selection of specific probiotic strains with targeted psychobiotic properties. Likewise, metabolomic analyses may help identify biochemical signatures—such as short-chain fatty acid profiles, tryptophan–kynurenine pathway metabolites, bile acids, or inflammatory mediators—that predict which patients are more likely to respond to FMT or psychobiotic interventions. These tools could eventually support the development of personalized microbiome-based treatment algorithms, transforming psychobiotics and FMT from generalized interventions into biomarker-guided therapies. It is envisioned that the progress in the field will rely on the integration of multi-omics data, computational modeling of the human microbiome, biomarkers for stratification of the population, and precision microbial engineering. This integration will eventually allow the transition from the general approach of probiotics/donor-based interventions to a personalized approach in the treatment of the human microbiome (Bautista et al., 2026).
